# Clinical Phenotypes, Treatment, and Outcomes of Rheumatic Immune–Related Adverse Events in Patients Treated With Immune Checkpoint Inhibitors: A Rheumatologists‐Referred Multicenter German Registry Cohort

**DOI:** 10.1002/acr2.90145

**Published:** 2026-09-08

**Authors:** Didzis Gailis, Fabian T.H. Ullrich, Sophia Dombret, Marc Schmalzing, Rebecca Hasseli‐Fräbel, Torsten Witte, Thea Thiele, Uta Kiltz, Mara Oleszowsky, Marcel Müller, Karolina Gente, Christof Specker, Matthias B. Thaler, Alla Skapenko, Hendrik Schulze‐Koops

**Affiliations:** ^1^ Division of Rheumatology and Clinical Immunology, Department of Medicine IV Ludwig‐Maximilians‐Universität (LMU) University Hospital, LMU Munich Munich Germany; ^2^ Department of Rheumatology and Clinical Immunology University Hospital Würzburg Würzburg Germany; ^3^ Department of Rheumatology and Clinical Immunology University Hospital Münster Münster Germany; ^4^ Department of Rheumatology and Immunology Hannover Medical School Hannover Germany; ^5^ Rheumazentrum Ruhrgebiet Herne Germany; ^6^ Rheumatology Private Practice Cologne Germany; ^7^ Institute for Medical Information Processing, Biometry and Epidemiology LMU University Hospital Munich Germany; ^8^ Department of Internal Medicine V (Hematology, Oncology and Rheumatology) Heidelberg University Hospital Heidelberg Germany; ^9^ Department of Rheumatology and Clinical Immunology Protestant Clinics Essen‐Mitte Essen Germany

## Abstract

**Objective:**

Immune checkpoint inhibitors (ICIs) induce a broad range of immune‐related adverse events (irAEs), including rheumatic manifestations. Evidence from dedicated rheumatology cohorts on the clinical spectrum, severity, treatment, and outcomes of rheumatic irAEs (rh‐irAEs) remains limited. We aimed to describe clinical phenotypes, treatment, and outcomes of rh‐irAEs in a national registry.

**Methods:**

The German ERIN Registry (Registry for the Documentation of Rheumatic Immunotherapy‐Related Adverse Events) is a multicenter observational registry of adults receiving ICIs. Rh‐irAEs were classified using a rheumatology‐oriented phenotype‐based framework. Variables included demographics, oncological characteristics, treatments, and outcomes. Severity was graded by Common Terminology Criteria for Adverse Events version 5.0. Univariate analyses identified predictors of second‐line systemic therapy.

**Results:**

As of January 1, 2026, 60 patients (56.7% men; median age 66 years) were included. Predominant malignancy was melanoma (40.0%). The leading phenotype was rheumatoid arthritis‐like polyarthritis (41.7%), followed by polymyalgia rheumatica‐like (18.3%) and peripheral spondyloarthritis‐like syndrome (11.7%). Axial spondyloarthritis (axSpA)–like syndrome occurred in younger patients. Rh‐irAEs were moderate or severe in 90%; 23.3% required hospitalization and 38.3% led to permanent ICI discontinuation. Systemic glucocorticoids (GCs) were used in 85.0%; 58.3% were GC‐refractory and 53.3% required second‐line immunomodulatory therapy. Complete remission was achieved in 33.3% and partial remission in 51.7%. Oncological progression occurred in 15.0% overall and in 28.1% of patients requiring second‐line therapy. Male sex was the strongest predictor of second‐line systemic therapy.

**Conclusion:**

Rh‐irAEs in a rheumatologists‐referred population were more severe than previously described, with frequent ICI discontinuation and escalation beyond GCs. Male sex was associated with treatment escalation. An underrecognized axSpA‐like phenotype occurred in younger patients, warranting further study.

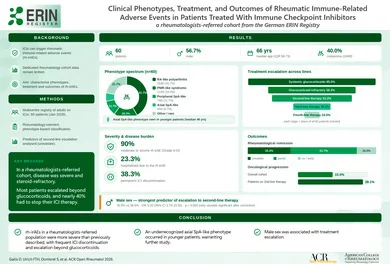

## INTRODUCTION

Immune checkpoint inhibitors (ICIs) have become a cornerstone of modern cancer immunotherapy. As of May 2025, the European Medicines Agency has approved more than 11 ICIs across more than 20 malignancies in the metastatic setting, with rapidly expanding indications in earlier disease stages—including the neoadjuvant setting—and with novel delivery routes such as subcutaneous formulations.^1^, ^2^ Eligibility for ICI therapy has expanded substantially, from 1.5% of cancer diagnoses in 2011 to 56.6% in 2023, with estimated overall response rates rising^3^ from 0.1% to 20.1%.

The expanding use of ICIs has important cross‐disciplinary implications, particularly for rheumatology, given the growing recognition of immune‐related complications. In addition to inducing de novo inflammatory syndromes, ICIs can exacerbate pre‐existing autoimmune diseases. Rheumatic immune–related adverse events (rh‐irAEs)—most commonly affecting the musculoskeletal system—were initially underreported in pivotal ICI trials but appear to be markedly more frequent in real‐world clinical practice, with prevalence estimates ranging from 1.5% to 22%. This wide range likely reflects both under‐recognition due to limited clinical familiarity and the often delayed onset of symptoms.^4^

A broad clinical spectrum has now been described, ranging from frequent manifestations (inflammatory arthritis, sicca syndrome, and polymyalgia rheumatica [PMR]–like syndrome) to rarer ones such as myositis, sarcoidosis‐like reaction, and vasculitides. These phenotypes differ substantially in severity and therapeutic requirements: some, such as myositis, carry life‐threatening risks and demand distinct management.

Management strategies for rh‐irAEs remain largely empirical owing to the absence of robust evidence‐based guidelines. In 2020, the EULAR published its Points to Consider for the diagnosis and management of rh‐irAEs, providing an initial conceptual framework^4^; however, these recommendations were necessarily based on expert consensus rather than randomized controlled trials. Although additional real‐world evidence has accumulated since then, systemic glucocorticoids (GCs) remain the foundation of therapy. In contrast to other irAEs such as colitis, rh‐irAEs often arise later in the treatment course and frequently require prolonged immunomodulation beyond GCs, raising concerns regarding their potential impact on oncologic outcomes in ICI‐treated patients.^5^, ^6^

To address this rapidly evolving gap in clinical evidence, the ERIN Registry (Registry for the Documentation of Rheumatic Immunotherapy‐Related Adverse Events) was established in March 2024 at the University Hospital Munich and subsequently expanded nationally. The ERIN Registry constitutes a dedicated, multicenter, practice‐oriented online database for the systematic documentation of rh‐irAEs associated with ICIs. Here, we report data on the first 60 patients included in the registry as of January 1, 2026.

## PATIENTS AND METHODS

### The registry

The ERIN Registry is a national, multicenter, prospective and retrospective observational online registry accessible to health care professionals across six German rheumatology centers. Data are recorded either longitudinally or as single entries.

### Inclusion criteria

Adult patients (≥18 years) with a rheumatologist‐confirmed diagnosis of a rh‐irAE occurring during or after ICI therapy.

### Exclusion criteria

Patients were excluded only if musculoskeletal symptoms were clearly attributable to alternative nonimmune causes (eg, trauma, infection, or metastatic disease) or if available clinical data were insufficient to confirm the diagnosis of rh‐irAE.

### Classification of rh‐irAEs

Treating rheumatologists documented rh‐irAEs using predefined clinical categories reflecting the primary presentation. For rheumatologically meaningful classification, these entries were subsequently re‐categorized according to clinical presentation and resemblance to established rheumatic disease phenotypes into the following groups: rheumatoid arthritis (RA)–like polyarthritis (predominantly symmetrical small‐joint involvement); peripheral spondyloarthritis (SpA)–like syndrome (large‐joint oligo‐ or polyarthritis with enthesitis and/or tenosynovitis); PMR‐like syndrome (inflammatory proximal muscle pain ± arthritis); axial SpA (axSpA)–like syndrome (predominantly axial involvement); noninflammatory arthralgia; activated osteoarthritis; sicca syndrome; and miscellaneous (myositis, sarcoidosis, vasculitis, and other rare presentations).

### Data collection

At each site, retrospective and prospective chart reviews used a standardized electronic case report form. Collected data include demographics, type and duration of ICI therapy, occurrence of rh‐irAEs and other irAEs, and detailed rh‐irAE characteristics (localization, systemic symptoms such as morning stiffness, and laboratory data including erythrocyte sedimentation rate, C‐reactive protein [CRP], antinuclear antibodies [ANA], rheumatoid factor, anticitrullinated protein antibodies, antineutrophil cytoplasmic antibodies, myositis‐specific antibodies), synovial fluid analysis, and HLA‐B27 status. Hospitalization was recorded as a yes or no variable and only when it occurred because of the rh‐irAE; inpatient admission and emergency‐department presentation were not distinguished.

Adverse events were graded according to Common Terminology Criteria for Adverse Events (CTCAE version 5.0; grade 1 = mild, 2 = moderate, 3 = severe, 4 = life‐threatening, and 5 = fatal). Treatments and responses were recorded from both oncologic and rheumatologic perspectives. Patients with pre‐existing autoimmune diseases were not excluded.

### Treatment of musculoskeletal irAEs

Therapeutic approaches included nonsteroidal anti‐inflammatory drugs (NSAIDs), intra‐articular and systemic GCs, colchicine, conventional synthetic disease‐modifying antirheumatic drugs (csDMARDs), biologic disease‐modifying antirheumatic drugs (bDMARDs), targeted synthetic disease‐modifying antirheumatic drugs, and other therapies (cyclophosphamide, intravenous immunoglobulin, and iloprost).

### Outcome assessment

Clinical outcome was categorized as complete remission (resolution of symptoms), partial remission (persistent symptoms with improvement), or no remission (persistent symptoms without improvement). In the absence of validated response criteria for rh‐irAEs, “improvement” reflected the treating rheumatologist's clinical assessment, and no quantitative minimum‐response threshold was applied. Follow‐up duration was calculated from the onset of the rh‐irAE to the last documented clinical presentation, and outcome status was ascertained at the last available assessment. Cancer response was evaluated according to Response Evaluation Criteria in Solid Tumours and categorized as complete response, partial response, stable disease, or progression.

### Statistical analysis

Descriptive statistics summarized demographic, clinical, laboratory, and treatment variables. Categorical variables are reported as absolute numbers and percentages, and continuous variables as medians with interquartile ranges (IQR).

Group comparisons were performed using Fisher's exact test for categorical variables. Univariate analyses evaluated associations between prespecified categorical variables (pre‐existing rheumatic disease, concomitant nonrheumatic irAEs, elevated CRP, male sex, melanoma as underlying malignancy, age ≥65 years, and combination ICI therapy) and escalation to second‐line systemic therapy, defined as initiation of any systemic treatment for the rh‐irAE beyond first‐line therapy, including csDMARDs, bDMARDs, GC re‐escalation, or iloprost; surgical intervention was not counted as escalation. Odds ratios (ORs) with 95% confidence intervals (CIs) were calculated. Owing to the limited sample size, no multivariable modeling was performed. To account for multiple comparisons across the seven variables tested, a Bonferroni‐corrected significance threshold of *P* < 0.007 (0.05 divided by 7 comparisons) was applied. Combination ICI therapy was defined as concurrent administration of two checkpoint inhibitors (nivolumab + ipilimumab, n = 11; nivolumab + relatlimab, n = 1).

All statistical analyses were performed using GraphPad Prism (version 10.0). Conventional data visualizations were generated in Prism; the Sankey diagram illustrating therapeutic trajectories was created using a custom Python script. Descriptive analyses were conducted in Microsoft Excel.

### Ethics and funding

The registry is funded exclusively through internal sources of the Division of Rheumatology and Clinical Immunology, Ludwig‐Maximilians‐Universität (LMU) University Hospital, without industry involvement. The protocol was approved by the ethics committee and data protection officer of the LMU Klinikum (project 23‐0407). Written informed consent was obtained from all participants.

## RESULTS

### Patient characteristics

A detailed summary of demographic and oncologic characteristics is provided in Table 1. Since its initiation and as of January 1, 2026, the ERIN Registry has enrolled 60 patients (n = 34 men, 56.7%), with a median age of 66 years (IQR 58–73). The most common underlying malignancy was malignant melanoma (n = 24, 40.0%), followed by non–small cell lung cancer (n = 9, 15.0%) and urothelial carcinoma (n = 7, 11.7%); the full distribution is provided in Table 1.

**Table 1:** Baseline demographic, oncologic, and treatment characteristics of patients included in the ERIN Registry (n = 60)*

Characteristic	Data
Demographics	
Sex, male, n (%)	34 (56.7)
Age, median (IQR), years	66 (58–73)
Age of axSpA‐like subgroup, median (IQR), years	46 (44–49.8)
Underlying malignancy, n (%)	
Malignant melanoma	24 (40.0)
NSCLC	9 (15.0)
Urothelial carcinoma	7 (11.7)
Breast carcinoma	5 (8.3)
RCC	5 (8.3)
SCC^a^	4 (6.7)
SCLC	2 (3.3)
DLBCL	1 (1.7)
CLL	1 (1.7)
Pleural mesothelioma	1 (1.7)
Angiosarcoma	1 (1.7)
ICI therapy administered, n (%)	
Pembrolizumab	21 (35.0)
Nivolumab	17 (28.3)
Combination nivolumab + ipilimumab	11 (18.3)
Atezolizumab	4 (6.7)
Durvalumab	3 (5.0)
Tislelizumab	1 (1.7)
Cemiplimab	1 (1.7)
Relatlimab	1 (1.7)
Avelumab	1 (1.7)
Prior treatment received, n (%)	
Targeted therapy	31 (51.7)
Chemotherapy	27 (45.0)
Radiotherapy	23 (38.3)

* axSpA, axial spondyloarthritis; CLL, chronic lymphocytic leukemia; DLBCL, diffuse large B cell lymphoma; ICI, immune checkpoint inhibitor; IQR, interquartile range; NSCLC, non–small cell lung cancer; RCC, renal cell carcinoma; SCC, squamous cell carcinoma; SCLC, small cell lung cancer.

^a^ Includes esophageal, cutaneous, and laryngeal SCCs.

### ICI therapy

Oncologic treatment characteristics are presented in Table 1. Most patients received programmed cell death‐1 inhibitors, predominantly pembrolizumab (n = 21, 35.0%) and nivolumab (n = 17, 28.3%). Combination nivolumab plus ipilimumab was administered in 18.3% (n = 11).

RA‐like polyarthritis and PMR‐like syndrome occurred across all ICI classes without therapy‐specific clustering. In contrast, combination ICI therapy was numerically over‐represented among peripheral SpA‐like (3 of 7 patients, 42.9%) and vasculitic presentations (2 of 3 patients, 66.7%) compared with its 18.3% representation in the overall cohort; this observation is descriptive and limited by small subgroup sizes.

Prior chemotherapy, targeted therapy, and radiotherapy were received by 45.0%, 51.7%, and 38.3% of patients, respectively. Two patients (3.3%) had received immunomodulatory treatment before ICI initiation. ICI therapy was temporarily interrupted in 9 patients (15.0%) and permanently discontinued in 23 patients (38.3%) due to the rh‐irAE.

### Pre‐existing rheumatic disease and de novo rh‐irAEs

A pre‐existing rheumatic disease was documented in seven patients (11.7%), including Sjögren's disease (n = 2; 3.3%), PMR (n = 2; 3.3%), psoriatic arthritis (PsA), giant cell arteritis (GCA), and axSpA (each n = 1; 1.7%). Accordingly, 53 patients (88.3%) developed de novo rh‐irAEs in the absence of any pre‐existing rheumatic disease, whereas 7 patients (11.7%) had a pre‐existing rheumatic disease, of whom 5 (8.3%) experienced a flare.

### Non–rh‐irAEs

Eighteen patients (30.0%) developed additional non–rh‐irAEs. The most frequently observed manifestations were pneumonitis (n = 6; 10.0%), hepatitis and colitis (each n = 3; 5.0%), and thyroiditis and nephritis (each n = 2; 3.3%).

### Latency from ICI initiation to rh‐irAE onset

An overview of differences according to time of onset and rh‐irAE is presented in Table 2. Median time from ICI initiation to rh‐irAE onset was 7.2 months (IQR 3.0–20.0). Latency was longest for peripheral SpA‐like syndrome, activated osteoarthritis, and RA‐like polyarthritis (median 12.9–13.7 months), shorter for PMR‐like (4.4 months) and axSpA‐like syndrome (4.8 months), and shortest for vasculitis (2.1 months).

**Table 2:** Latency of rh‐irAEs by clinical phenotype*

rh‐irAE phenotype	n	Median, months	IQR
All rh‐irAEs	60	7.2	3.0–20.0
RA‐like polyarthritis	25	12.9	6.9–26.4
PMR‐like syndrome	11	4.4	2.8–6.2
Peripheral SpA‐like syndrome	7	13.7	5.6–22.2
axSpA‐like syndrome	4	4.8	3.9–8.2
Vasculitis (GCA or GPA)	3	2.1	1.9–66.6
Activated osteoarthritis	3	13.7	10.3–16.4
Arthralgia (noninflammatory)	2	5.0	3.6–6.5
Monoarthritis	2	4.3	3.7–5.0

* Median time from initiation of immune checkpoint inhibitor therapy to onset of rh‐irAEs is shown in months. Phenotypes represented by a single case (myositis, sicca syndrome, sarcoidosis‐like reaction) were excluded due to limited interpretability. Latency estimates for rare phenotypes should be interpreted cautiously. axSpA, axial spondyloarthritis; GCA, giant cell arteritis; GPA, granulomatosis with polyangiitis; IQR, interquartile range; PMR, polymyalgia rheumatica; RA, rheumatoid arthritis; rh‐irAEs, rheumatic immune–related adverse events; SpA, spondyloarthritis.

### Clinical presentation of rh‐irAEs

A detailed overview of phenotypes, associated symptoms, and severity grades is provided in Table 3. The most frequent phenotype was RA‐like polyarthritis (n = 25; 41.7%), followed by PMR‐like syndrome (n = 11; 18.3%) and peripheral SpA‐like syndrome (n = 7; 11.7%). axSpA‐like syndrome occurred in four patients (6.7%), and activated osteoarthritis in three (5.0%). Arthralgia and monoarthritis were each observed in two patients (3.3%). Vasculitic manifestations were observed in three patients (5.0%; one GCA, one unspecified vasculitis, and one GPA‐like syndrome). Further rare manifestations included sarcoidosis‐like reaction, sicca syndrome, and myositis (each n = 1; 1.7%). Patients with axSpA‐like syndrome were substantially younger than the overall cohort, with a median age of 46 years (IQR 44–49.8).

**Table 3:** Clinical spectrum, associated symptoms, and severity of rh‐irAEs according to Common Terminology Criteria for Adverse Events (n = 60)*

rh‐irAE phenotype	n (%)	Flare, n (%)	Arthralgia, n (%)	Myalgia, n (%)	Morning stiffness, n (%)	Grade 1, n (%)	Grade 2, n (%)	Grade 3, n (%)	Grade 4, n (%)
RA‐like polyarthritis	25 (41.7)	0 (0.0)	25 (100.0)	8 (32.0)	17 (68.0)	3 (12.0)	15 (60.0)	7 (28.0)	0 (0.0)
PMR‐like syndrome	11 (18.3)	2 (18.2)	8 (72.7)	9 (81.8)	6 (54.5)	1 (9.1)	6 (54.5)	4 (36.4)	0 (0.0)
Peripheral SpA‐like syndrome	7 (11.7)	1 (14.3)	7 (100.0)	2 (28.6)	3 (42.9)	0 (0.0)	4 (57.1)	3 (42.9)	0 (0.0)
axSpA‐like syndrome	4 (6.7)	1 (25.0)	1 (25.0)	2 (50.0)	3 (75.0)	0 (0.0)	2 (50.0)	2 (50.0)	0 (0.0)
Vasculitis (GCA or GPA)	3 (5.0)	1 (33.3)	2 (66.7)	2 (66.7)	0 (0.0)	0 (0.0)	0 (0.0)	3 (100.0)	0 (0.0)
Activated osteoarthritis	3 (5.0)	0 (0.0)	3 (100.0)	0 (0.0)	2 (66.7)	0 (0.0)	2 (66.7)	1 (33.3)	0 (0.0)
Monoarthritis	2 (3.3)	0 (0.0)	2 (100.0)	1 (50.0)	1 (50.0)	0 (0.0)	1 (50.0)	1 (50.0)	0 (0.0)
Arthralgia (noninflammatory)	2 (3.3)	0 (0.0)	2 (100.0)	1 (50.0)	0 (0.0)	0 (0.0)	2 (100.0)	0 (0.0)	0 (0.0)
Myositis	1 (1.7)	0 (0.0)	0 (0.0)	0 (0.0)	0 (0.0)	0 (0.0)	0 (0.0)	0 (0.0)	1 (100.0)
Sicca syndrome	1 (1.7)	0 (0.0)	0 (0.0)	0 (0.0)	0 (0.0)	0 (0.0)	1 (100.0)	0 (0.0)	0 (0.0)
Sarcoidosis‐like reaction	1 (1.7)	0 (0.0)	0 (0.0)	0 (0.0)	0 (0.0)	0 (0.0)	0 (0.0)	0 (0.0)	1 (100.0)
Total	60 (100.0)	5 (8.3)	50 (83.3)	25 (41.7)	32 (53.3)	4 (6.7)	33 (55.0)	21 (35.0)	2 (3.3)

* Phenotypes are classified according to predominant clinical presentation. Myalgia and morning stiffness are reported as associated symptoms. Severity grading follows Common Terminology Criteria for Adverse Events version 5.0. Flare is defined as reactivation of a pre‐existing rheumatic disease of the same entity under ICI therapy. axSpA, axial spondyloarthritis; GCA, giant cell arteritis; GPA, granulomatosis with polyangiitis; PMR, polymyalgia rheumatica; RA, rheumatoid arthritis; rh‐irAEs, rheumatic immune–related adverse events; SpA, spondyloarthritis.

Morning stiffness and nonspecific myalgias were reported by 32 (53.3%) and 25 (41.7%) patients, respectively, with phenotype‐specific distributions detailed in Table 3. By CTCAE grading, rh‐irAEs were predominantly moderate (grade 2, 55.0%) or severe (grade 3, 35.0%); grade 4 events occurred in two patients (myositis and sarcoidosis‐like reaction). Phenotype‐specific severity distributions are detailed in Table 3.

### Laboratory and immunologic findings

At the time of rh‐irAE diagnosis, CRP elevation was present in 44 patients (73.3%) and leukocytosis in 15 patients (25.0%); no inflammatory laboratory abnormalities were detected in 14 patients (23.3%). Exact CRP concentrations are not recorded in the registry and individual laboratory‐specific cut‐offs were used.

ANA were positive in 15 patients (25.0%). The predominant immunofluorescence patterns were fine‐granular (n = 4, 26.7%), nucleolar (n = 3, 20.0%), and mixed granular–nucleolar or homogeneous (n = 2, 13.3%). In two patients (13.3%), ANA positivity was accompanied by SS‐A and/or SS‐B antibodies, which were already present before ICI initiation, consistent with a pre‐existing Sjögren's‐like serologic profile.

### Synovial and histopathologic findings

Diagnostic arthrocentesis confirmed an inflammatory joint effusion in nine patients (15.0%), with synovial fluid leukocyte cell counts of 6,200 to 15,300 cells/μL (median ≈ 11,000). Biopsies were performed in three patients (5.0%). One transbronchial needle aspiration revealed a granulomatous reaction in the drainage area of a tumor, consistent with a sarcoidosis‐like reaction, without evidence of malignancy. Two synovial biopsies showed active inflammatory changes, including one with high‐grade synovitis^7^ (Krenn score 5 = 2 + 2 + 1).

### Treatment

Hospitalization due to rh‐irAEs occurred in 14 patients (23.3%), with the highest subgroup rates for vasculitis (2 of 3), peripheral SpA‐like syndrome (2 of 7), and PMR‐like syndrome (3 of 11).

Intra‐articular GC injections were administered in 13 patients (21.7%) for local control of articular inflammation. Systemic GCs were initiated in 51 patients (85.0%) at a median initial dose of 20 mg prednisone equivalent/day (range 5–1,000), the highest being a single case of myositis requiring pulse therapy. Among phenotypes with multiple cases, RA‐like polyarthritis and PMR‐like syndrome were typically treated with a median 20 mg/day; phenotype‐specific dosing is detailed in Table 4.

**Table 4:** Treatment of rh‐irAEs by clinical phenotype*

Phenotype	n (%)	Hosp., n (%)	NSAIDs, n (%)	i.a. GC, n (%)	Systemic GC (first‐line), n (%)	Dose, median (IQR), mg	GC‐refractory, n (%)	csDMARDs, n (%)	bDMARDs, n (%)
RA‐like polyarthritis	25 (41.7)	4 (16.0)	5 (20.0)	6 (24.0)	23 (92.0)	20 (20–40)	16 (64.0)	10 (40.0)	5 (20.0)
PMR‐like syndrome	11 (18.3)	3 (27.3)	2 (18.2)	1 (9.1)	10 (90.9)	20 (10–45)	9 (81.8)	4 (36.4)	1 (9.1)
Peripheral SpA‐like syndrome	7 (11.7)	2 (28.6)	0 (0.0)	3 (42.9)	6 (85.7)	40 (7.5–80)	6 (85.7)	4 (57.1)	3 (42.9)
axSpA‐like syndrome	4 (6.7)	1 (25.0)	4 (100.0)	1 (25.0)	3 (75.0)	10 (10–40)	1 (25.0)	1 (25.0)	1 (25.0)
Vasculitis (GCA or GPA)	3 (5.0)	2 (66.7)	0 (0.0)	0 (0.0)	3 (100.0)	40 (20–60)	1 (33.3)	0 (0.0)	0 (0.0)
Activated osteoarthritis	3 (5.0)	0 (0.0)	3 (100.0)	1 (33.3)	1 (33.3)	40 (40–40)	1 (33.3)	0 (0.0)	0 (0.0)
Monoarthritis	2 (3.3)	0 (0.0)	0 (0.0)	1 (50.0)	1 (50.0)	20 (0–40)	1 (50.0)	2 (100.0)	0 (0.0)
Arthralgia (noninflammatory)	2 (3.3)	0 (0.0)	0 (0.0)	0 (0.0)	2 (100.0)	35 (10–60)	0 (0.0)	0 (0.0)	0 (0.0)
Myositis	1 (1.7)	1 (100.0)	0 (0.0)	0 (0.0)	1 (100.0)	1,000	0 (0.0)	0 (0.0)	1 (100.0)
Sicca syndrome	1 (1.7)	0 (0.0)	0 (0.0)	0 (0.0)	0 (0.0)	–	0 (0.0)	0 (0.0)	0 (0.0)
Sarcoidosis‐like reaction	1 (1.7)	1 (100.0)	0 (0.0)	0 (0.0)	1 (100.0)	40 (40–40)	0 (0.0)	0 (0.0)	0 (0.0)
Total	60 (100.0)	14 (23.3)	14 (23.3)	13 (21.7)	51 (85.0)	20 (10–22.5)	35 (58.3)	21 (35.0)	11 (18.3)

* For each phenotype, the table summarizes patient numbers, hospitalization frequency, use of NSAIDs, intra‐articular GCs, systemic GCs (including median initial dose with IQR), GC‐refractory disease, and the proportion requiring csDMARDs or bDMARDs as second‐line therapy. Specific therapeutic agents used as second‐ and third‐line therapy are detailed in Supplementary Table S1. axSpA, axial spondyloarthritis; bDMARD, biologic disease‐modifying antirheumatic drug; csDMARD, conventional synthetic disease‐modifying antirheumatic drug; GC, glucocorticoid; GCA, giant cell arteritis; GPA, granulomatosis with polyangiitis; i.a., intra‐articular; IQR, interquartile range; NSAID, nonsteroidal anti‐inflammatory drug; PMR, polymyalgia rheumatica; RA, rheumatoid arthritis; rh‐irAEs, rheumatic immune–related adverse events; SpA, spondyloarthritis.

Systemic GCs were administered for a median duration of 4.1 months (mean 8.6 months; range 0.6–46.3); longest durations occurred in RA‐like polyarthritis (up to 26 months) and PMR‐like syndrome (up to 14 months), whereas vasculitic, sarcoidosis‐like, and GPA cases received short‐term high‐dose courses (<4 months). In 12 patients (20.0%), GC therapy was ongoing at data cut‐off.

GC‐refractory disease occurred in 35 patients (58.3%), and second‐line csDMARD or bDMARD therapy was initiated in 32 (53.3%). Methotrexate was most frequently used, followed by tumor necrosis factor (TNF) and interleukin‐6 (IL‐6) inhibitors. Three patients received GC re‐escalation, one with vasculitis received iloprost, and one underwent total knee arthroplasty for severe activated osteoarthritis. A detailed overview of phenotype‐specific treatment strategies, including hospitalization, NSAIDs, intra‐articular and systemic GCs, and second‐line therapy, is provided in Table 4.

In the univariate analysis, male sex was the strongest predictor of escalation to second‐line systemic therapy (76.5% vs 38.5%; OR 5.20, 95% CI 1.70–15.92; *P* = 0.004), the only variable meeting the Bonferroni‐corrected threshold (*P* < 0.007). Pre‐existing inflammatory rheumatic disease showed a nominal association with a lower probability of escalation (OR 0.09, 95% CI 0.01–0.77; *P* = 0.013) that did not survive correction and should be regarded as hypothesis‐generating. Combination ICI therapy showed a numerically higher escalation likelihood without reaching significance (OR 4.23; *P* = 0.10). None of the other assessed variables—elevated CRP, concomitant nonrheumatic irAEs, melanoma, and age ≥65 years—reached statistical significance (Table 5). In a sensitivity analysis restricted to de novo rh‐irAEs (n = 53), the association between male sex and escalation was unchanged (83.3% vs 43.5%; OR 6.50, 95% CI 1.83–23.04; *P* = 0.004).

**Table 5:** Factors associated with escalation to second‐line systemic therapy in patients with rheumatic irAEs*

Variable	Cohort, n (%)	Escalation, n (%)	OR (95% CI)	*P* value
Pre‐existing rheumatic disease	7 (11.7)	1 (14.3)	0.09 (0.01–0.77)	0.013
Concomitant nonrheumatic irAEs	18 (30.0)	11 (61.1)	1.07 (0.35–3.31)	1.000
Elevated CRP	44 (73.3)	28 (63.6)	1.75 (0.48–6.37)	0.383
Sex, male	34 (56.7)	26 (76.5)	5.20 (1.70–15.92)	0.004
Melanoma as underlying malignancy	24 (40.0)	15 (62.5)	1.19 (0.41–3.43)	0.794
Age ≥65 years	32 (53.3)	18 (56.3)	0.71 (0.25–2.02)	0.603
Combination ICI therapy	12 (20.0)	10 (83.3)	4.23 (0.85–21.10)	0.100

* Univariate analysis was performed using Fisher's exact test. Data are presented as absolute numbers with corresponding percentages. ORs are shown with 95% CIs. A Bonferroni‐corrected threshold of *P* < 0.007 (0.05/7) was applied for the seven comparisons. Only the association with male sex (*P* = 0.004) met this threshold; the association with pre‐existing rheumatic disease did not survive correction and is reported as a hypothesis‐generating signal. No significant associations were observed for concomitant non–rheumatic irAEs, elevated CRP, melanoma as underlying malignancy, age ≥ 65 years, or combination ICI therapy. Escalation was defined as initiation of any systemic treatment for the rheumatic irAE beyond first‐line therapy (csDMARDs, bDMARDs, GC re‐escalation, or iloprost; n = 36); surgical intervention was not counted. CI, confidence interval; bDMARD, biologic disease‐modifying antirheumatic drug; CRP, C‐reactive protein; csDMARD, conventional synthetic disease‐modifying antirheumatic drug; GC, glucocorticoid; ICI, immune checkpoint inhibitor; irAE, immune‐related adverse event; OR, odds ratio.

Of the 32 patients receiving second‐line therapy, 12 (37.5%) switched to another agent due to insufficient response or intolerance. Three additional patients received a combination DMARD or intra‐articular GCs, resulting in 15 patients (25.0% of the cohort) receiving third‐line therapy overall. The most frequently used third‐line agent was adalimumab (5 of 15, 33.3%) (Table 4).

**Figure 1:**
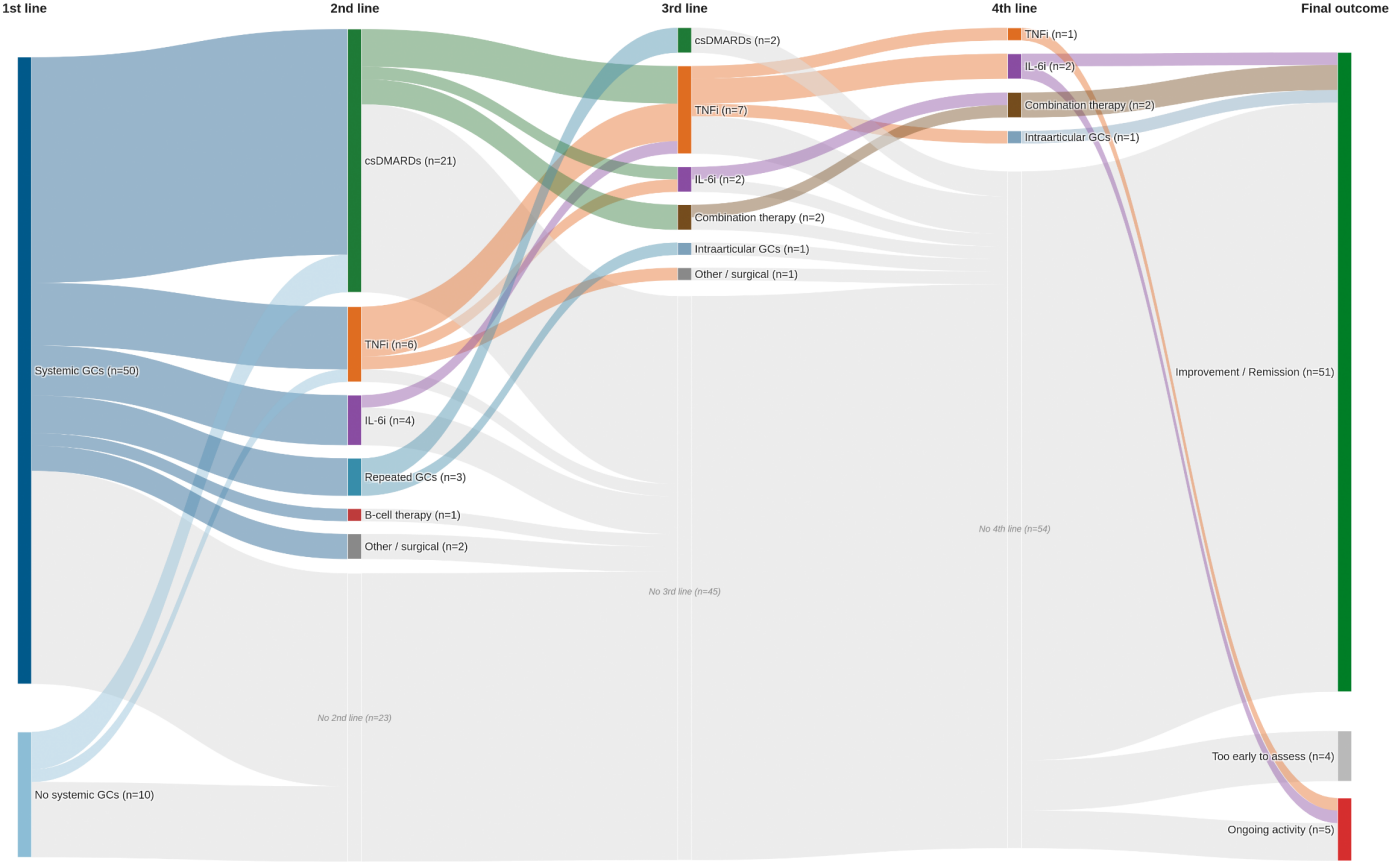
Sankey diagram of therapeutic trajectories in patients with rheumatic immune–related adverse events. The diagram illustrates treatment pathways and clinical outcomes across consecutive treatment lines, beginning with first‐line therapy (predominantly systemic GCs) and progressing through second‐, third‐, and fourth‐line immunomodulatory strategies. Treatment categories include systemic GCs, csDMARDs, TNFi, IL‐6i, B cell therapy, intra‐articular GCs, combination disease‐modifying antirheumatic drug therapy, and other or surgical interventions. Stream width is proportional to the number of patients transitioning between treatment steps. Final nodes indicate clinical outcomes, categorized as improvement or remission, ongoing disease activity, or cases too early to assess at time of data cut. csDMARD, conventional synthetic disease‐modifying antirheumatic drug; GC, glucocorticoid; IL‐6i, interleukin‐6 inhibitor; TNFi, tumor necrosis factor inhibitor.

Among the 15 patients requiring third‐line therapy, primary nonresponse was the leading reason for escalation (n = 8; 53.3%), followed by intolerance (n = 3; 20.0%) and oncologic progression (n = 2; 13.3%). In single cases, treatment modification occurred due to secondary loss of efficacy or patient‐initiated discontinuation (each n = 1; 6.7%). Six patients (10.0% of the total cohort; 17.1% of steroid‐refractory cases) required escalation to a fourth‐line immunomodulatory therapy. Figure 1 illustrates the therapeutic trajectories of patients with rh‐irAEs across successive lines of treatment.

### Oncologic outcomes

In the overall cohort, nine patients (15.0%) experienced oncologic progression at any point during follow‐up—seven during second‐line and two during third‐line immunomodulatory therapy; none progressed during first‐line GC therapy alone. Partial or complete remission was achieved in 9 patients (15.0%), and 40 (66.7%) maintained stable disease. Response was undocumented in two patients (3.3%). Among the nine patients with oncologic progression, six were men and three women, reflecting the cohort's male predominance.

Among the 32 patients receiving csDMARDs or bDMARDs as second‐line or later‐line therapy, oncologic progression occurred in 9 (28.1%): under methotrexate in 4, adalimumab in 4, and etanercept in 1. Given the small sample, heterogeneity of malignancies, and nonrandomized allocation, no conclusions can be drawn about differential agent effects on tumor outcomes.

Rh‐irAE severity showed no clear relationship with oncologic outcome, with substantial overlap between groups. GC duration was longer in patients with progression (median 7.2 vs 5.1 months), whereas initial doses were comparable (20 vs 21.25 mg).

### Rheumatologic outcomes

Complete remission was achieved in 20 patients (33.3%) and partial remission in 31 patients (51.7%). No remission was documented in five patients (8.3%). In three patients (5.0%), the outcome was too early to assess at the time of data cut‐off, and in one patient (1.7%), it was not known. The median follow‐up from rh‐irAE onset to last documented presentation was 9.9 months (IQR 4.1–31.1; range 0.2–74.4). Five patients (8.3%) died during follow‐up, none directly attributed to rh‐irAEs (three tumor progression, one infectious, and one unknown).

## DISCUSSION

Our registry provides a comprehensive characterization of the clinical spectrum, management strategies, and outcomes of rh‐irAEs. The demographic and oncologic profile of the ERIN cohort aligns with established rh‐irAE registries such as the Canadian Research Group of Rheumatology in Immuno‐Oncology cohort,^8^ supporting the external validity of our findings. One notable feature of ERIN is the particularly high share of melanoma among underlying malignancies. Rather than indicating a higher intrinsic susceptibility of melanoma patients to rh‐irAEs, this likely reflects the referral pathways into our registry: our close collaboration with the international Side Effect Registry Immuno‐Oncology at the LMU Department of Dermatology and our participation in monthly interdisciplinary toxicity boards may facilitate early referral of melanoma patients with musculoskeletal complaints to rheumatology. More broadly, the malignancy distribution in rh‐irAE registries reflects prescribing and referral structures rather than true prevalence.

Patients with axSpA‐like syndrome were substantially younger than the overall cohort, suggesting age‐related immunologic distinctions between rh‐irAE phenotypes. Several factors complicate the diagnosis of an axSpA‐like manifestation in this setting: axial imaging must be interpreted against degenerative, posttraumatic, and oncologic change; HLA‐B27 was negative in three of four patients; and classification criteria developed for primary SpA do not transfer to ICI‐associated disease. Delineating this entity will require larger cohorts and standardized axial imaging.

Given the heterogeneity of published classification systems for rh‐irAEs, the ERIN Registry was deliberately designed using a rheumatology‐specific, phenotype‐based framework to enhance clinical applicability. In a systematic review of 69 publications, Ghosh et al demonstrated marked classification inconsistency.^9^ Consistent with the approaches proposed by Kostine et al and Gómez‐Puerta et al, we applied a clinically oriented framework aligned with accepted disease entities.^10^, ^11^

PsA‐like syndromes were reclassified into a broader peripheral SpA category to include patients with clinically predominant enthesitis and/or tenosynovitis with or without peripheral arthritis, even in the absence of psoriasis. Establishing a unified classification system will be essential to improve comparability across studies.

RA‐like polyarthritis represented the most common phenotype (~42%). This contrasts with earlier large datasets—such as the 927‐patient cohort reported by Melia et al—in which noninflammatory manifestations (arthralgia and myalgia) predominated, whereas inflammatory rh‐irAEs were identified in only 3.9% of patients.^12^ These discrepancies likely reflect tertiary‐referral bias, with academic rheumatology centers mainly evaluating complex inflammatory cases, whereas milder manifestations often remain unrecognized. Indeed, only 4 of 12 patients with rheumatic irAEs were evaluated by a rheumatologist in one prior study.^13^ A recent whole‐body magnetic‐resonance‐imaging study demonstrated that ICI‐exposed patients with arthralgia frequently exhibit inflammatory lesions comparable to those in inflammatory arthritis, suggesting substantially higher true prevalence of inflammatory rh‐irAEs.^14^

Patients with established rheumatic autoimmune diseases were generally excluded from ICI trials; therefore, real‐world safety data remain limited. A systematic review of 23 studies by Liu et al^15^ reported that patients with pre‐existing rheumatic disease—particularly RA—may experience disease flares following ICI therapy.

In our registry, seven patients (11.7%) had pre‐existing inflammatory rheumatic disease; of these, five experienced a flare, and notably none had underlying RA. Furthermore, pre‐existing rheumatic disease was not significantly associated with escalation to second‐line systemic therapy; given the small number of affected patients, this observation is hypothesis‐generating and requires prospective validation, ideally stratified by underlying condition, because risk may differ between entities.

Although rh‐irAEs are well known to occur more frequently under combination ICI therapy, few studies have systematically examined the timing of symptom onset across distinct rheumatic phenotypes. In our cohort, the overall median latency was 7.2 months. PMR‐like and axSpA‐like syndromes occurred earlier, whereas vasculitis emerged particularly early (median 2.1 months). In contrast, peripheral SpA‐like syndrome and osteoarthritis‐like flares exhibited longer latency. These findings should be interpreted cautiously given the small subgroup sizes but broadly align with a prospective observational study from three centers in Spain (n = 73), except for PMR‐like syndrome, which occurred later in that cohort.^11^

A central therapeutic priority is to maintain ICI therapy whenever oncologically feasible, as emphasized by the EULAR Points to Consider. In our cohort, ICI therapy had to be permanently discontinued in nearly 40% of patients and temporarily interrupted in an additional 15%—substantially exceeding rates in most published cohorts (eg, 15% permanent discontinuation in Gómez‐Puerta et al^11^) and underscoring the urgent need for strategies to sustain cancer immunotherapy despite rheumatic toxicity.

Compared with other retrospective cohorts, patients in our registry experienced a notably more severe clinical course. Approximately 90% of rh‐irAEs were categorized as moderate or severe. Systemic GCs were initiated in over 80% of cases, with a median starting dose of 20 mg—substantially exceeding the <10 mg recommended by the EULAR Points to Consider.

Strikingly, 53.3% of patients required immunomodulatory therapy beyond GCs, including csDMARDs (predominantly methotrexate, 35.0%) and bDMARDs (18.3%). In contrast, in the cohort reported by Melia et al^12^ (n = 118), no patient received a bDMARD and only 16 were treated with methotrexate. Similarly, in the cohort reported by Gómez‐Puerta et al,^11^ only 11% required DMARD therapy and none received biologics. The German MalheuR cohort reported an intermediate picture, with bDMARD use of 10.0% in patients with underlying melanoma and 12.2% in those with non–small cell lung cancer. The substantially higher bDMARD rate in ERIN may reflect our rheumatology‐based recruitment, which enriches for more severe, treatment‐refractory disease. These differences may indicate that the therapeutic demand of rh‐irAEs, particularly in a rheumatologists‐referred setting, is greater than previously reported.

Although numerous studies have identified risk factors for rh‐irAE development, far fewer have investigated determinants of severity. The prospective CanRIO cohort (139 patients) reported no meaningful age‐related differences in rh‐irAE frequency or severity—consistent with our findings.^16^ Additional studies identified osteoarthritis, body mass index, and smoking as independent risk factors for irAE‐arthritis.^17^ Braaten et al demonstrated in a prospective observational cohort that tender joint counts, duration of ICI exposure, combination therapy, and the presence of additional irAEs were associated with persistence of inflammatory arthritis.^18^ Although outcome definitions differ—persistence of arthritis versus escalation to second‐line systemic therapy in the present analysis—male sex emerged as the strongest predictor of treatment escalation in our cohort (76.5% vs 38.5% in females). Consistent with the Braaten cohort, elevated CRP levels were not significantly associated with treatment escalation in the present analysis. However, the registry currently captures CRP only as a categorical variable, limiting assessment of dose–response relationships and preventing definition of clinically meaningful thresholds. Future registry iterations should incorporate quantitative CRP values, tender joint counts, and candidate biomarkers such as serum calprotectin^19^ and T cell activation signatures.^20^

The influence of steroid‐sparing immunomodulators, particularly TNF and IL‐6 inhibitors, on tumor control remains uncertain. A recent meta‐analysis highlighted that most studies assessing anti‐TNF therapy focused on ICI colitis, involved limited infliximab exposure, and were restricted by retrospective design and immortal‐time bias; similar limitations apply to IL‐6 receptor inhibitor studies.^21^ Within these constraints, available data are reassuring.^21^, ^22^, ^23^ A discordant signal comes from Bass et al, who reported a higher rate of cancer progression with TNF inhibitors versus tocilizumab in ICI‐associated arthritis, although limited by confounding by indication and small sample sizes.^24^

In the prospective, multicenter study by Gente et al, male sex, flares of pre‐existing rheumatic disease, and prolonged GC exposure were associated with unfavorable oncologic outcomes, whereas DMARD treatment appeared to exert a protective effect.^25^ In our ERIN cohort, oncologic progression occurred in 15.0% of patients overall and in 28.1% of those requiring second‐ or later‐line DMARD therapy. Owing to the limited number of events and the heterogeneity of underlying malignancies and immunomodulatory agents, drug‐specific risk estimates cannot yet be derived. These figures are subject to confounding by indication: escalation was driven by more severe, steroid‐refractory disease, and such patients more often had their ICI interrupted—both of which independently raise the risk of progression. These descriptive data therefore do not permit a causal interpretation. Initial GC doses were comparable between patients with and without progression; however, patients with progression received GCs for a longer duration, an observation that should be interpreted cautiously.

Validated activity scores or remission criteria for rh‐irAEs are currently lacking. We pragmatically defined complete remission as the absence of clinical symptoms, achieved in 33% of cases. However, the category “improved” encompasses a broad spectrum of partial responses, underscoring the urgent need for standardized, phenotype‐specific response criteria.

Our study has limitations inherent to its retrospective, registry‐based design. As a referral‐based registry, ERIN captures only patients referred to and confirmed in rheumatology; the unreferred or unconfirmed proportion is unknown and center‐dependent, precluding population‐level incidence estimates. Reporting bias may compound this, as early‐stage registry data favor severe or unusual cases. Both mechanisms enrich the cohort for complex disease. Measurement is likewise constrained: diagnostic work‐up varied across centers; CTCAE grading may insufficiently capture the subjective burden and functional impairment associated with rh‐irAEs; biomarker data, particularly quantitative CRP, remain incomplete; hospitalization could not be resolved into admission versus emergency presentation, and at some centers short inpatient stays for rheumatologic work‐up form part of routine care irrespective of severity; and partial remission was defined without a quantitative threshold, with outcome ascertained at a variable single time point rather than at a fixed interval. Finally, sample sizes within several phenotypic subgroups are small; in particular, the seven patients with pre‐existing rheumatic disease (one escalation event) preclude a formal comparison of flares versus de novo rh‐irAEs.

Nevertheless, the ERIN Registry highlights several unmet needs, including broader recruitment across rheumatology and oncology centers, standardized classification and response criteria, systematic biomarker collection, and long‐term follow‐up—all essential to refine phenotype‐specific management, assess the safety of advanced immunomodulation, and identify predictors of severe or refractory disease.

## AUTHOR CONTRIBUTIONS

All authors contributed to at least one of the following manuscript preparation roles: conceptualization AND/OR methodology, software, investigation, formal analysis, data curation, visualization, and validation AND drafting or reviewing/editing the final draft. As corresponding author, Dr Gailis confirms that all authors have provided the final approval of the version to be published and takes responsibility for the affirmations regarding article submission (eg, not under consideration by another journal), the integrity of the data presented, and the statements regarding compliance with institutional review board/Declaration of Helsinki requirements.

## Supporting information


**Disclosure Form**:

**Supplementary Table S1.** Therapeutic agents used as second‐ and third‐line immunomodulatory therapy in patients with rheumatic immune‐related adverse events (rh‐irAEs). For each agent, the number of patients receiving it as second‐line therapy is shown (n = 32). The ‘Continued’ column reflects patients who remained on their second‐line agent at data cut‐off. The ‘Switched to next‐line agent’ column reflects discontinuation of the second‐line agent in favour of a different therapy. The ‘Third‐line’ column shows the total number of patients receiving each agent as third‐line therapy (n = 15). The difference between 12 discontinuations and 15 total third‐line patients is accounted for by patients who continued their second‐line agent in combination with an additional third‐line therapy (combination regimens are listed as such). Three additional patients received glucocorticoid re‐escalation and one patient with vasculitis received iloprost as second‐line therapy; these are reported in the main text and not included in this table.
