# Supplementary material for: Clinical Phenotypes, Treatment, and Outcomes of Rheumatic Immune–Related Adverse Events in Patients Treated With Immune Checkpoint Inhibitors: A Rheumatologists‐Referred Multicenter German Registry Cohort
Source: ACR Open Rheumatol. 2026 Sep 8;8(9):e90145. doi: 10.1002/acr2.90145 (PMC13552557; doi:10.1002/acr2.90145)
Supplement: Supplementary file 1 [file ACR2-8-e90145-s001.docx]

**Supplementary Table S1.** Therapeutic agents used as second- and third-line immunomodulatory therapy in patients with rheumatic immune-related adverse events (rh-irAEs). For each agent, the number of patients receiving it as second-line therapy is shown (n = 32). The ‘Continued’ column reflects patients who remained on their second-line agent at data cut-off. The ‘Switched to next-line agent’ column reflects discontinuation of the second-line agent in favour of a different therapy. The ‘Third-line’ column shows the total number of patients receiving each agent as third-line therapy (n = 15). The difference between 12 discontinuations and 15 total third-line patients is accounted for by patients who continued their second-line agent in combination with an additional third-line therapy (combination regimens are listed as such). Three additional patients received glucocorticoid re-escalation and one patient with vasculitis received iloprost as second-line therapy; these are reported in the main text and not included in this table.

| **Therapeutic agent** | **Second-line (n=32) n (%)** | **Continued n (%)** | **Switched to next-line agent n (%)** | **Third-line (n=15) n (%)** |
| --- | --- | --- | --- | --- |
| Methotrexate | 20 (62.5) | 14 (70.0) | 6 (30.0) | 2 (13.3) |
| Adalimumab | 4 (12.5) | 0 (0.0) | 4 (100.0) | 5 (33.3) |
| Tocilizumab | 3 (9.4) | 2 (66.7) | 1 (33.3) | 2 (13.3) |
| Infliximab | 1 (3.1) | 1 (100.0) | 0 (0.0) | 1 (6.7) |
| Etanercept | 1 (3.1) | 0 (0.0) | 1 (100.0) | – |
| Sarilumab | 1 (3.1) | 1 (100.0) | 0 (0.0) | – |
| Rituximab | 1 (3.1) | 1 (100.0) | 0 (0.0) | – |
| Leflunomide | 1 (3.1) | 1 (100.0) | 0 (0.0) | – |
| Golimumab | – | – | – | 1 (6.7) |
| Ixekizumab | – | – | – | 1 (6.7) |
| Methotrexate + adalimumab | – | – | – | 1 (6.7) |
| Sulfasalazine + methotrexate | – | – | – | 1 (6.7) |
| Intra-articular glucocorticoids | – | – | – | 1 (6.7) |
| **Total** | **32 (100.0)** | **20 (62.5)** | **12 (37.5)** | **15 (100.0)** |

*Abbreviations:* GC = glucocorticoid; rh-irAEs = rheumatic immune-related adverse events.
